# CRISPRi targeting to the *Neisseria gonorrhoeae* R-loop or G4 mutant region restores pilin antigenic variation

**DOI:** 10.1128/mbio.01347-26

**Published:** 2026-07-06

**Authors:** Selma Metaane, H. Steven Seifert

**Affiliations:** 1 Department of Microbiology-Immunology, Feinberg School of Medicine, Northwestern University 12244, Chicago, Illinois, USA; University of California, Berkeley, Berkeley, California, USA

**Keywords:** *Neisseria*, pilus, antigenic variation, CRISPRi, R-loop, G-quadruplex

## Abstract

*Neisseria gonorrhoeae* pilin antigenic variation (pilin Av) is a complex diversity generation system. Pilin Av depends on a Rec-dependent gene conversion event initiated by an R-loop (*gar* sRNA) and a resultant G-quadruplex (G4) DNA structure. Mutations of the *garP* promoter or the G4-forming sequence each prevent pilin Av. We targeted a Type I-C CRISPR interference (CRISPRi) complex to the mutant *garP* or G4 loci, and CRISPRi targeting to a specific location on the leading strand restored pilin Av. In contrast, targeting the CRISPRi complex to other locations on either the leading or lagging strand was lethal. The CRISPRi restoration of pilin Av depended on the standard recombination factors, confirming the conserved pathway. Inverting the *garP-G4* region confirmed that the target strand dictates viability and Av outcome. Together, these results reveal that we can replace the R-loop and resultant G4 structure by specific CRISPRi targeting, providing insights into nucleic acid, secondary structure-dependent genome dynamics.

**IMPORTANCE:**

*Neisseria*’s pilin antigenic variation remains one of the most sophisticated DNA diversification processes in bacteria, yet the trigger that initiates recombination remains unclear. Here, we show that targeting a Type I CRISPR interference complex is sufficient to restore pilin variation in strains unable to form an R-loop or a G-quadruplex structure. Our findings reveal that the position and strand of R-loop formation, rather than its precise sequence context, determine whether cells undergo antigenic variation, remain viable, or die. This establishes a new framework in which localized topological stress, not a particular DNA structure, plays a role in *Neisseria gonorrhoeae* pilin antigenic variation.

## INTRODUCTION

The human-restricted pathogenic bacterium *Neisseria gonorrhoeae* (gonococcus, Gc) is the main causative agent of gonorrhea. Gc Type IV pilus is a primary colonization and virulence factor that is highly variable, allowing Gc to evade immune surveillance. The pilus major subunit is the pilin protein PilE, encoded by the *pilE* gene (1–4) that undergoes a gene conversion event called pilin antigenic variation (Pilin Av) (5, 6). Gc Pilin Av is mediated by the donation of portions of one of the ~19 *pilS* silent copies (1) into the variable region of the *pilE* gene. Pilin Av depends on standard recombination and repair factors, including RecA, RecOR, and partially on RecJ and RecQ (7–12).

Pilin Av requires the formation of two nucleic acid structures upstream of the *pilE* gene. An RNA:DNA hybrid consisting of the *gar* small RNA-mediated R-loop, allowing a G-quadruplex (G4) structure to form (13). The promoter of the *gar* sRNA (*garP*) is about 300 bp upstream of the *pilE* open reading frame and is adjacent to the G4-forming sequence (14). When transcription of a *cis*-acting *gar* sRNA is initiated (15, 16), RNA polymerase unwinds the DNA, inducing the formation of a *gar* R-loop that subsequently permits G4 folding (14).

Although both the R-loop and the G-quadruplex are required for pilin Av, the precise molecular mechanisms underlying the gene conversion event remain unanswered. The reasons these structures have a major role in the process are unknown. R-loops can have dual outcomes; they contribute to regulated functions under physiological conditions, and they can lead to genome instability when dysregulated or improperly resolved (17). Previous studies showed that pilin Av does not occur if the *garP* and *gar* region is inverted on the same strand or is switched from the leading strand to the lagging strand (14, 18). Because R-loops and G-quadruplexes can interfere with replication (19), the prevailing model for pilin Av initiation posits that replication forks stall at G4 and R-loop structures, initiating gene conversion at *pilE* (13).

Gc does not express any CRISPR systems, while the closely related organism *Neisseria meningitidis* expresses a Type II Cas9 CRISPR (20, 21). Recently, a *Neisseria lactamica* Type I-C CRISPR-Cas3 cascade was structurally and mechanistically characterized, revealing how the multi-subunit Cascade complex recognizes PAM-containing targets, forms a full R-loop, and remains stably associated with DNA independently of Cas3-mediated cleavage (22). This *N. lactamica* Type I-C Cascade was subsequently adapted as a Gc programmable CRISPR interference (CRISPRi) tool by omitting *cas3*, allowing sequence-specific transcriptional repression without DNA degradation (23). In this system, Cascade binding involves not only the formation of a crRNA-DNA heteroduplex but also extensive protein-DNA contacts, including PAM recognition by Cas8 and stabilization of the displaced non-target DNA strand along Cas11 subunits, resulting in a highly stable nucleoprotein complex (22). When targeted to promoter-proximal PAM sites, the persistent Cascade-DNA assembly sterically impairs RNA polymerase access and elongation, leading to effective gene knockdown (23). We made the surprising finding that targeting the Gc CRISPRi system to the G4 and R-loop locus in loss-of-function mutants rescues Gc pilin Av but can also be a lethal event.

## RESULTS

### CRISPRi targeting to the G4-R-loop locus has different phenotypes

**Fig 1:**
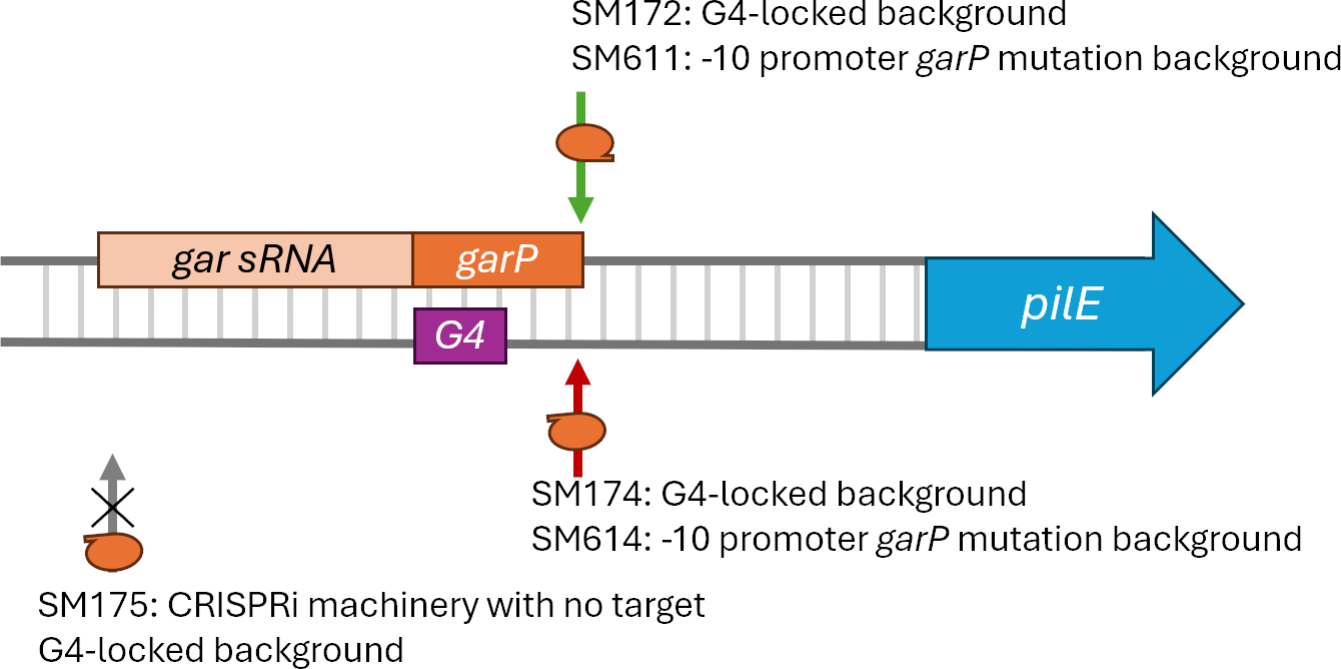
Schematic representation of the *garP*-G4 locus showing the positions of the *garP* promoter, the G4-forming sequence, and the PAM sites targeted by the CRISPRi guides used in strains SM172, SM174, SM611, and SM614, as well as the non-targeting control strain SM175.

**Fig 2:**
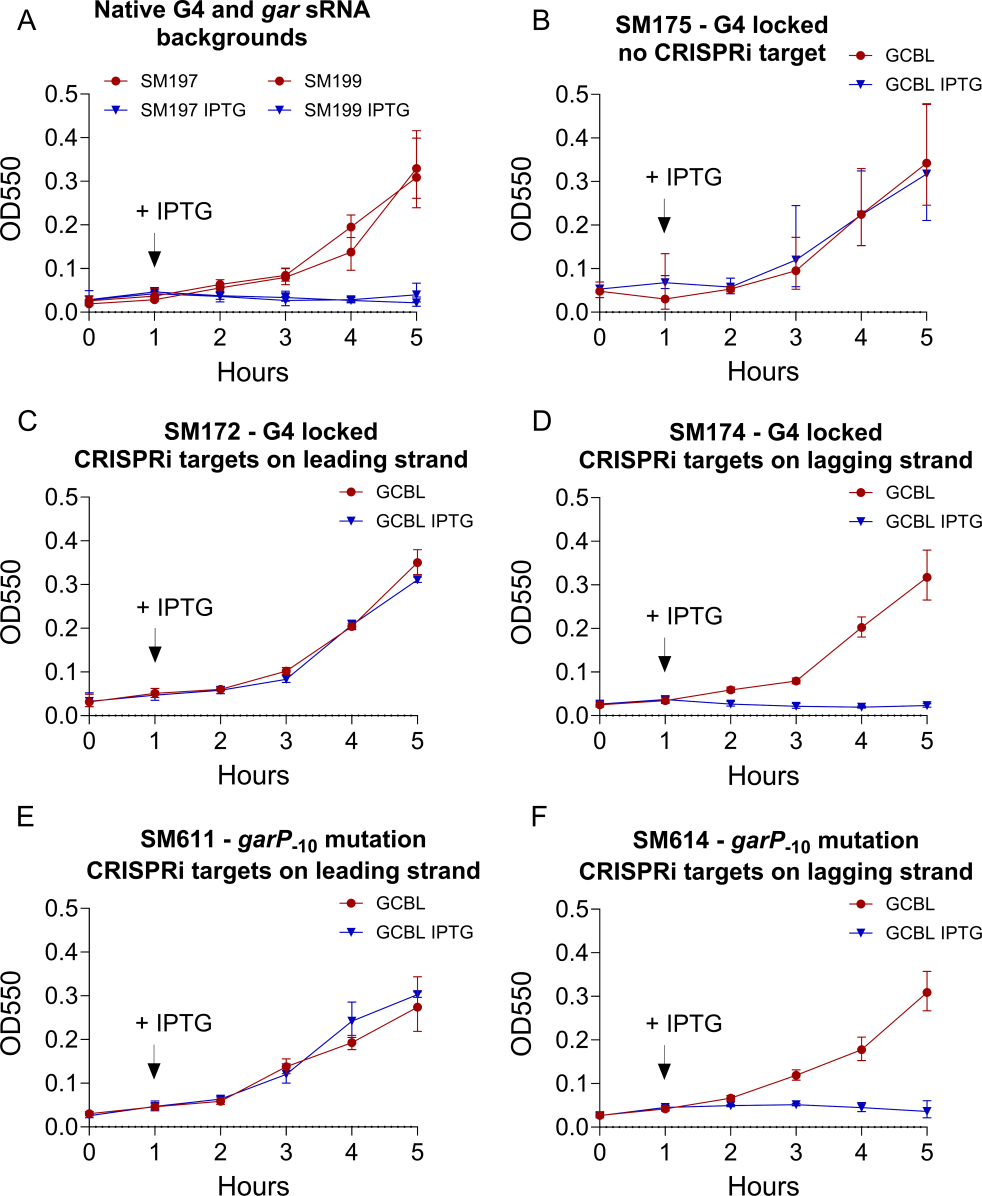
CRISPRi-mediated targeting of the *garP-*G4 region produces strain-specific lethality. Growth curves of each strain in gonococcal base liquid (GCBL) medium with or without IPTG induction (black arrow), which activates the Type I-C CRISPRi machinery. (**A**) Growth curves of strains SM197 and SM199, which both carry native G-quadruplex (G4) and *gar* sequences. Strain SM197 expresses a CRISPRi guide targeting the PAM site on the leading strand, whereas strain SM199 carries a guide targeting the PAM site on the lagging strand. (**B**) Control strain SM175, lacking a CRISPRi target, exhibits comparable growth with or without IPTG in the growth media. (**C**) Strain SM172 (G4-locked, guide targeting the leading strand) shows normal growth under CRISPRi induction. (**D**) Strain SM174 (G4-locked, guide targeting the lagging strand) displays synthetic lethality in the presence of IPTG. (**E**) Strain SM611 (*garP*_-10_ promoter mutant, guide targeting the leading strand) grows normally in the presence of IPTG. (**F**) Strain SM614 (*garP*_-10_ promoter mutant, guide targeting the lagging strand) fails to grow when CRISPRi was induced. Each data point represents the mean ± SD of three independent biological replicates.

The *gar* transcript forms an R-loop on the C-rich strand, allowing the G4 structure to form on the G-rich strand (13, 16). We asked whether we could target the CRISPRi machinery to inhibit *gar* transcription and subsequently pilin Av. The two PAM sites upstream of the *gar* promoter (*garP*) are located on opposite strands (Fig. 1). We therefore directed the CRISPRi machinery to the *garP* region using guide RNAs targeting these two PAM sites. Upon isopropyl β-D-1-thiogalactopyranoside (IPTG) induction, constructs targeting either strand were lethal (Fig. 2A). Because the DNA-CRISPRi complex, together with the physiological R-loop and G4 structures, is likely difficult to resolve, we next tested the CRISPRi machinery in R-loop/G4 deficient mutants.

When we targeted the CRISPRi machinery to the two PAM sites in a G4 mutant (designated G4-locked) (16) or a *garP*_-10_ mutant (13), we observed a strand-dependent difference in viability upon CRISPRi induction (Fig. 2). Targeting the CRISPRi machinery to G4-forming sequence on the lagging strand was lethal in both the G4-locked (SM174) and the *garP*_-10_ mutant (SM614) (Fig. 2D and F). In contrast, when we targeted the CRISPRi to the PAM site located at the leading strand, both the G4-locked strain (SM172) and the *garP*_-10_ mutant (SM611) showed normal growth, similar to that observed without IPTG CRISPRi induction or with a non-targeting CRISPRi-negative control with no guide RNA (Fig. 2B, C and E). Notably, under CRISPRi induction conditions, we observed the emergence of SM174 escape mutants. We isolated four independent clones that were able to grow despite CRISPRi targeting. One of these clones carried a deletion of *pilE* that extended upstream into the region containing the G4-*gar* locus, whereas the three others harbored mutations affecting components of the CRISPRi machinery. These results indicate that the selective pressure imposed by CRISPRi targeting can promote the selection of escape variants. To determine whether the phenotype observed under CRISPRi induction was associated with restoration of native *gar* transcription, we quantified *gar* sRNA levels by digital quantitative reverse transcription PCR (qRT-PCR) after 3 h of induction. In G4-locked strains, which display detectable *gar* expression in the absence of induction (Fig. S1A and B), CRISPRi reduced *gar* transcript levels to near the limit of detection. Similarly, in the *gar*_-10_ promoter mutant, where *gar* transcription is already minimal, *gar* transcripts remained undetectable upon CRISPRi induction (Fig. S1C and D), regardless of the targeted strand. These results indicate that the observed phenotype occurs independently of *gar* sRNA production.

### Lethal CRISPRi targeting is associated with a localized loss of genomic integrity

**Fig 3:**
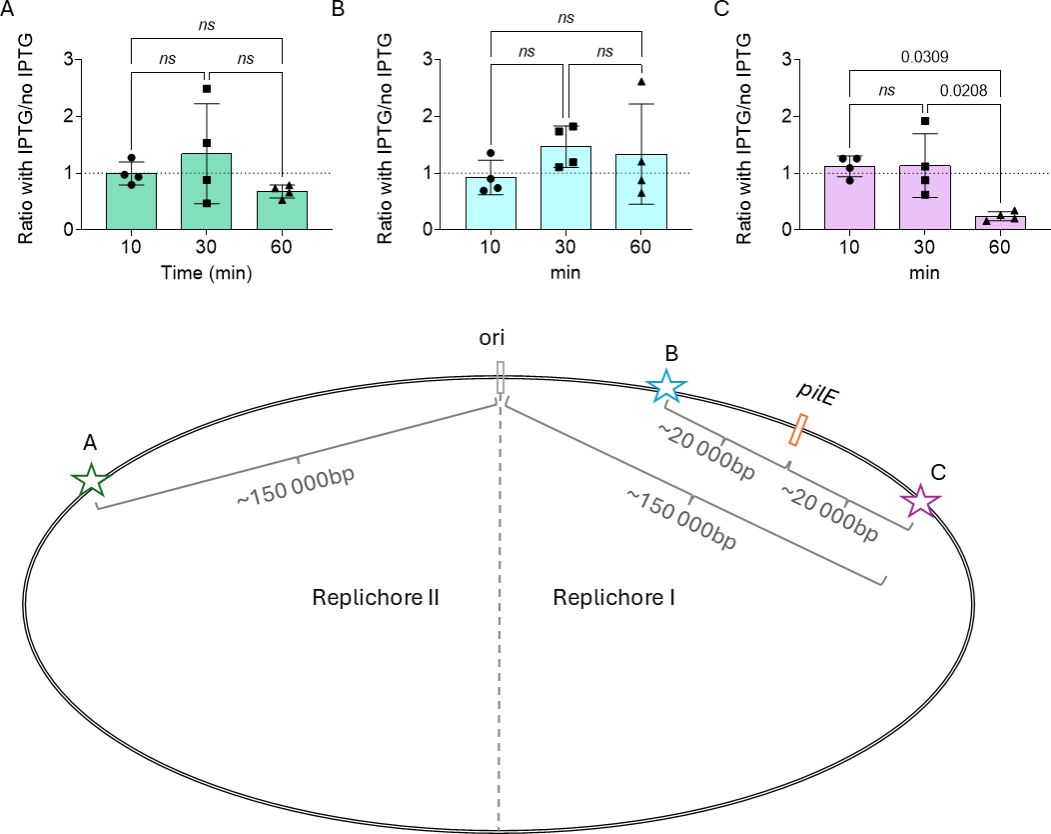
Time-dependent genomic copy number imbalance downstream of *pilE* upon CRISPRi induction. We measured relative genomic DNA abundance by digital PCR (dPCR) in the G4-locked strain SM174 carrying an IPTG-inducible lagging-strand-targeting CRISPRi. The cells were grown in gonococcal base liquid (GCBL) with or without IPTG for 10, 30, or 60 min. Three chromosomal loci were quantified and expressed as IPTG/non-IPTG ratios: (**A**) a distal locus on replichore II (~150 kb from the origin); (**B**) an intermediate locus on replichore I ~ 200 kb upstream of *pilE*; and (**C**) a locus located ~200 kb downstream of *pilE,* (~150 kb from the origin [schematized below]). Statistical analysis was performed using one-way analysis of variance. Each point represents an independent biological replicate; bars indicate mean ± SD. The dashed line denotes a ratio of 1.

Because CRISPRi targeting of the lagging strand in the G4-locked strain SM174 is lethal upon induction, we asked whether CRISPRi lethality was associated with detectable alterations in DNA content. We quantified chromosomal DNA abundance by digital PCR (dPCR) at defined loci surrounding *pilE* following CRISPRi induction. A complete round of Gc chromosome replication and cell division requires approximately 60 min (24). Accordingly, we sampled DNA copy number at 10 and 30 min after CRISPRi induction. Cultures from the same preculture were grown with or without IPTG. We quantified relative DNA abundance at three loci: locus A, located on replichore II (Fig. 3), lies approximately 150 kb from the origin of replication, as does locus C on replichore I, and these loci would replicate at similar times. We expressed DNA content as IPTG/non-IPTG ratios, a normalization that both controls for differences in template abundance and minimizes background variability arising from asynchronous chromosome replication. At locus A, we did not detect any change in relative DNA abundance at any time point (Fig. 3A). In contrast, locus C, located ~20 kb downstream of the CRISPRi binding site within the *gar-pilE* region, exhibited a significant decrease in genomic copy number after 60 min of CRISPRi induction (Fig. 3C). To more precisely localize this effect, we also quantified DNA abundance at locus B, which is ~200 kb upstream of the *gar-pilE* region, but we did not observe any significant change in locus abundance (Fig. 3B). Together, these results indicate that CRISPRi targeting of the lagging strand of the *garP-G4* region causes a localized decrease in DNA abundance downstream of *pilE*.

### CRISPRi targeting to the *garP* on the leading strand produces *pilE* variants

**Fig 4:**
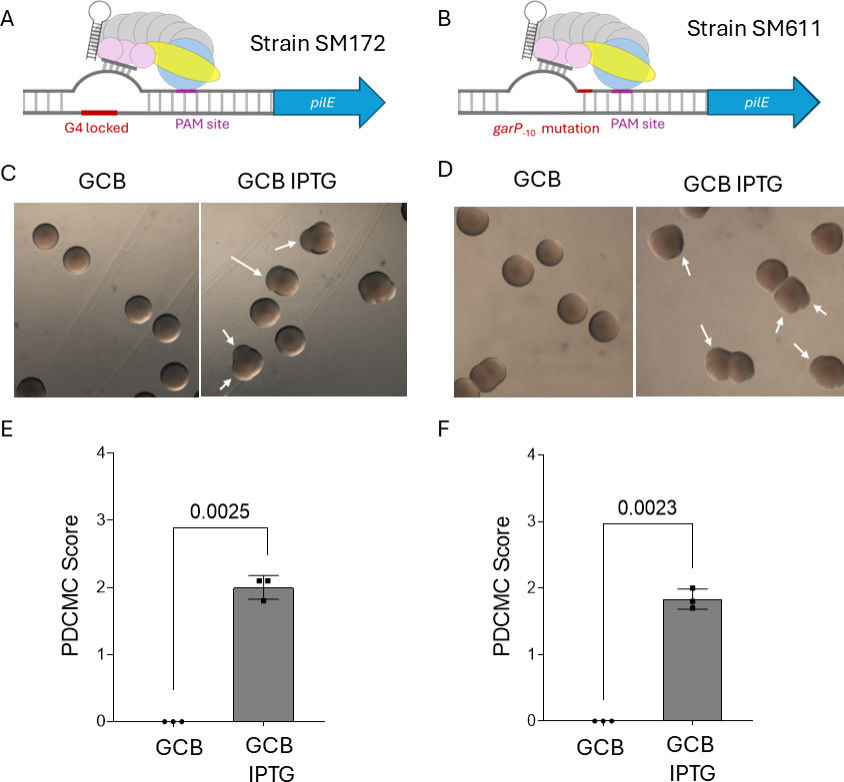
Strand-specific CRISPRi targeting of *garP* differentially affects viability and pilin antigenic variation. (**A and B**) Schematic representations of the CRISPRi-targeted *garP* region, (**C and D**) colony morphology after 30 h growth on gonococcal base (GCB) agar in the absence or presence of 1 mM IPTG, (**E and F**) pilus-dependent colony morphology change (PDCMC) scores (**A–C**) G4-locked strain SM172, in which CRISPRi targets the *garP* region on the leading strand, displays under-piliated colony morphotypes (blebs, white arrows) upon IPTG induction. (**B–D**) *garP*_-10_ mutant strain SM611, with CRISPRi targeting the leading strand, produces blebbed colonies (white arrows) upon IPTG induction, similar to the parent strain SM172. (**E**) Upon IPTG CRISPRi induction, we detected an increase in the PDCMC score in the SM172 G4-locked mutant. (**F**) Upon IPTG CRISPRi induction, we detected an increase in the PDCMC score in the SM611 *garP*_-10_ mutant. Data are presented as mean ± SD of three independent biological replicates; statistical significance was assessed using a Student’s *t*-test.

When pilin Av occurs within a colony, it generates phase-variant subpopulations exhibiting lower levels of piliation that eventually outgrow the parental colony; these non- or under-piliated variants are called blebs, and their occurrence is a surrogate measure of pilin Av. When we targeted CRISPRi to the leading strand, we observed blebs at the edges of the G4-locked strain (SM172) and the *garP*_-10_ mutant (SM611) colonies that are consistent with pilin Av (Fig. 4) (13). We performed the pilus-dependent colony morphology change (PDCMC) assay (8) for strains SM172 and SM611 to quantify pilin Av. As expected, there was no pilin Av as measured by PDCMC with no IPTG in the growth medium (Fig. 4C and D). After 30 h of growth under IPTG induction, we measured a PDCMC score consistent with the restoration of pilin Av (Fig. 4E and F).

**Fig 5:**
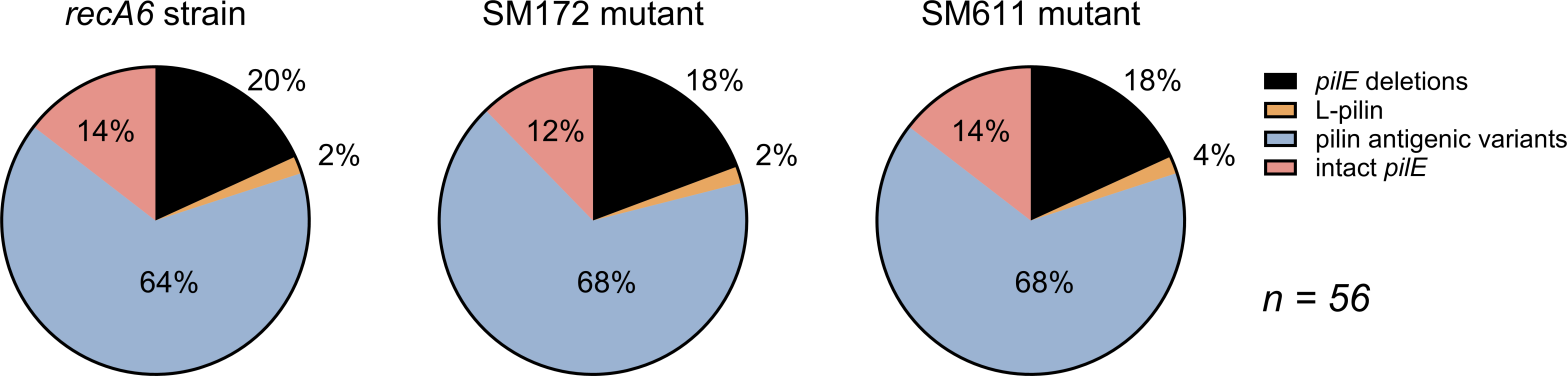
Distribution of *pilE* sequence type in the parental, SM172, and SM611 strains. Pie charts show the proportions of *pilE* sequence types contained in individual bleb isolates following RecA or CRISPRi induction. For each strain, sequences were categorized as pilin antigenic variants, intact *pilE*, *pilE* deletions, or L-pilin. The *recA6* strain yielded 64% antigenic variants, 14% intact *pilE*, 20% deletions, and 2% L-pilin. SM172 showed 68% antigenic variants, 12% intact *pilE*, 18% deletions, and 2% L-pilin. SM611 displayed 68% antigenic variants, 14% intact *pilE*, 15% deletions, and 3% L-pilin. A total of *n* = 56 sequences were analyzed across the three strain backgrounds.

Because colony morphology is an indirect surrogate for pilin antigenic variation, we directly determined the *pilE* sequence of individual CRISPRi-induced blebs. To provide a reference, we compared these variants with those obtained from a *recA6* strain, in which we induced pilin antigenic variation by IPTG-mediated RecA expression (25). The distribution of *pilE* variants was comparable among the *recA6* strain, the SM172 mutant, and the SM611 mutant in the presence of IPTG (Fig. 5). In each strain, most *pilE* sequences from the blebs were pilin antigenic variants (64% in the parental strain, 68% in SM172, and 68% in SM611). *pilE* deletions accounted for 20%, 18%, and 18%, respectively, consistent with previously published data (26, 27). Unchanged *pilE* alleles were 14% in the parental strain, 12% in SM172, and 14% in SM611, presumably *pilC* phase variants (28). We detected L-pilin sequences at low proportions (2% in the parental strain, 2% in SM172, and 4% in SM611). These analyses show that CRISPRi restoration of pilin antigenic variation is similar to the canonical pathway.

The analysis of *pilE* variant sequences showed that the regions undergoing sequence changes mapped to the expected semi-variable and hypervariable regions of *pilE*, which are the established sites of gene conversion (Fig. S2). Within these regions, the sequence tracts could be aligned to specific *pilS* silent copies, allowing the identification of likely donor loci contributing to each recombinant variant. The lengths and positions of the imported segments varied among isolates, consistent with the mosaic structures generated during pilin Av. Together, these observations show that the CRISPRi-induced variants arise through a gene conversion process, involving homologous recombination between *pilE* and the *pilS* repertoire, rather than through an alternative mechanism. The distinct distribution of recombination tracts compared to normal R-loop- and G4-dependent antigenic variation suggests that, while CRISPRi engages the canonical recombination machinery, it may influence the regional pattern of sequence exchange within *pilE*.

### CRISPRi-induced pilin variation uses standard recombination factors

**Fig 6:**
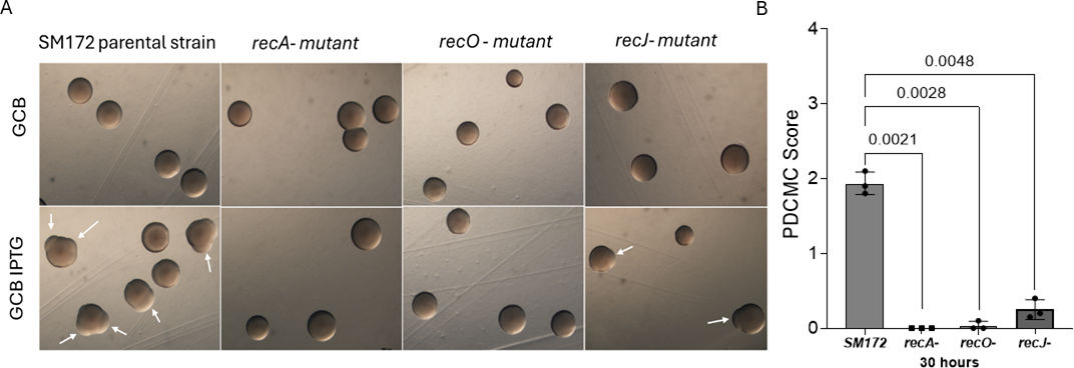
CRISPRi-induced pilin Av depends on standard recombination factors. (**A**) The parental strain SM172 and the SM172 *recA*, *recO*, and *recJ* mutants were grown on GCB with and without IPTG. Blebs were identified on the parental strain SM172 colonies on IPTG (white arrows) and some colonies of *recJ* but not on the colonies from *recA* or *recO* mutants. (**B**) Pilin antigenic variation levels measured by the surrogate PDCMC assay upon IPTG. Data are presented as mean ± SD of three independent biological replicates; statistical significance was assessed using a Student’s *t*-test to compare the *rec*- mutants to the SM172 parental strain.

To determine whether the CRISPRi-induced pilin variants used the same recombination factors as normal pilin Av, we tested strains with loss-of-function mutations in *recA*, *recO*, and *recJ*, gene products critical for pilin Av (9, 11, 12, 29). SM172 CRISPRi-induced blebs and PDCMC were absent in the *recA* or *recO* mutants (Fig. 6A and B). Moreover, the *recJ* mutant showed an intermediate level of pilin Av, similar to that reported for a *recJ* mutant (12, 30). These results show that the same *rec*-pathways mediate CRISPRi-induced pilin variation as the standard pilin Av process.

### Strand targeting, not G4 orientation, determines CRISPRi outcomes

**Fig 7:**
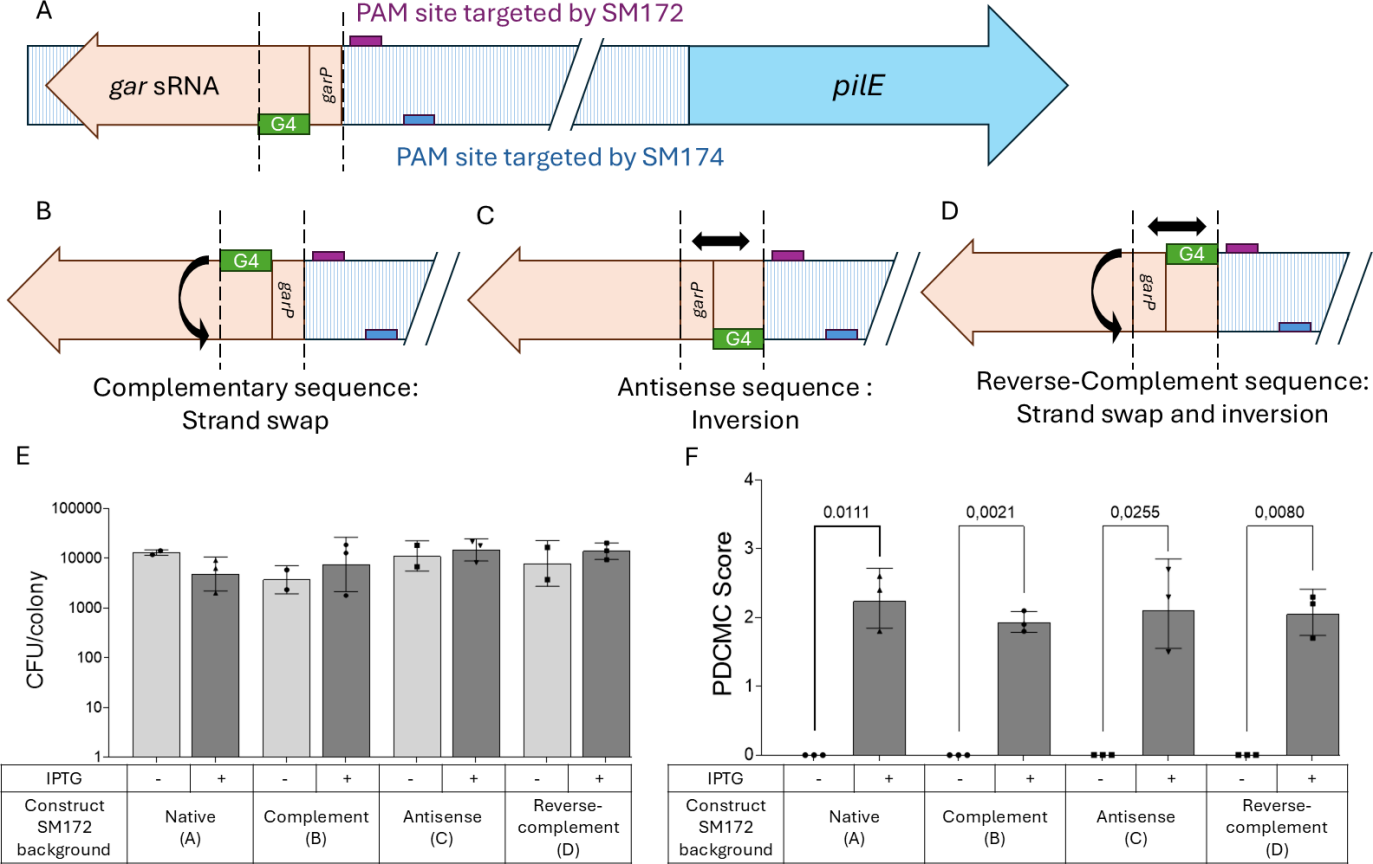
Configurations of the *garP*-G4 region in the tested constructs with the PAM sites targeted by the CRISPRi machinery. (**A**) Native configuration of the *garP*-G4 locus. In the original sequence, the PAM site targeted by guide SM172 is located on the leading strand (dark purple), whereas the PAM site targeted by SM174 is positioned on the lagging strand (blue). The dark orange rectangle represents the *gar* sRNA, and the green rectangle represents the G-quadruplex-forming sequence. (**B**) The *garP*-G4 region is replaced by its complement. Although the G4-forming sequence is preserved in this arrangement, it is unknown whether an R-loop can still form in this orientation. (**C**) The *garP*-G4 region is replaced with its antisense sequence, disrupting the gar sRNA sequence and therefore its associated R-loop. (**D**) Reverse-complement configuration, in which both the orientation and complementarity differ from the native arrangement, repositioning the targeted PAM site relative to the G4 motif. (**E**) Growth of SM172 constructs on solid media; CFU/colony were measured in the presence or absence of 1 mM IPTG after 30 h of growth. (**F**) Pilin antigenic variation levels in SM172 measured by the surrogate PDCMC assay. Data points are presented as mean ± SD of three independent biological replicates; statistical significance was assessed using a Student’s *t*-test, and only statistically significant differences are displayed.

Because targeting CRISPRi to the leading strand produced viable Gc, whereas targeting the lagging strand was lethal, we wondered whether this was due to the G4 and R-loop locations on these strands or to their orientation relative to the CRISPRi machinery. To address this question, we created strains in which the *garP*-G4 region was inverted or switched to the opposite strand in both orientations (Fig. 7A and B).

With all constructs, induction of the CRISPRi targeting the lagging strand was lethal, regardless of the orientation or configuration of the *garP*-G4 region (Fig. S3). These results indicate that CRISPRi targeting of the lagging strand is not tolerated at this locus. As a control, we measured the growth of a previously published strain (23) in which the CRISPRi machinery targets seven sites across the genome, three on the lagging strand, and four on the leading strand; targeting to all sites inhibits transcription (Fig. S4). Induction of CRISPRi did not impair growth, indicating that lagging-strand targeting *per se* is not lethal. SM172-derived strains carrying *garP*-G4 strand swap, inverted sequence, or both, remained viable upon IPTG induction and displayed pilin antigenic variation levels comparable to those observed in the parental SM172 strain (Fig. 7F). These results demonstrate that the orientation and strand identity of the *garP*-G4 sequence itself is not important for CRISPRi-induced pilin Av. Thus, neither the orientation nor the sequence identity of the *garP*-G4 region dictates CRISPRi-induced outcomes. Instead, strand identity at the site of Cascade binding is the dominant determinant of strain viability or the restoration of pilin variation.

## DISCUSSION

Using a catalytically inactive Type I-C CRISPRi system, we show that targeting the mutant *garP* and G4 region restores pilin Av in strains that are otherwise pilin Av-deficient. In many organisms, the presence of an R-loop and/or a G-quadruplex interferes with replication fork progression (19, 31). The prevailing model of pilin Av proposes that the native R-loop and the resultant G4 structure initiate pilin antigenic variation by inducing a stall or stop of the replication apparatus (14–16). This hypothesis is consistent with the catalytically inactive CRISPRi Cascade complex creating a physical barrier and also interrupting replication. Consistent with this model, our genomic copy-number analysis provides evidence of a localized perturbation in chromosome maintenance upon CRISPRi targeting of the lagging strand. Digital PCR revealed a time-dependent reduction in the abundance of genomic sequences located downstream of the CRISPRi binding site near the *garP-pilE* region, while loci at comparable distances from the origin on the opposite replichore or upstream of the target site remained unaffected. This pattern is indicative of localized genomic instability associated with CRISPRi binding, consistent with impaired replication or maintenance of chromosomal regions distal to a persistent protein-DNA obstacle.

Importantly, our results further show that neither the strand nor the orientation of the G4 is critical for allowing CRISPRi to rescue pilin Av or affect Gc viability. Inverting the sequences or switching the strand of the *garP*-G4 region results in cell viability and antigenic variation phenotypes compared with the parental strains SM172 and SM174. These data suggest the strand of the G4 and R-loop, or the DNA-CRISPRi complex, is a critical aspect of this complex diversity generation system. Why CRISPRi binding is tolerated on the leading strand but not on the lagging strand remains unclear. One might predict that replication-Cascade collisions on the leading strand would be more deleterious to chromosome integrity. It is possible that the five-protein CASCADE complex inhibits Okazaki fragment processing, but we presently do not have any data to support this hypothesis

Sequencing of under-piliated colony morphotypes confirms that CRISPRi-induced variants correspond to *pilE* gene conversion events rather than deletions, and the strict dependence of this process on *recA* and *recO* indicates that CRISPRi-induced variation proceeds through the normal homologous recombination pathway. This result suggests that CRISPRi restores pilin Av via the existing recombination process, rather than via a distinct pathway. Under physiological conditions, the G4 stabilizes the *gar* R-loop, such that both structures are required for pilin antigenic variation. However, the fact that the stably bound CRISPRi complex restores variation only when targeted to the leading strand bound to the *gar* sRNA suggests that formation of a strand-specific R-loop is a key determinant of antigenic variation. This conclusion is consistent with previous studies showing that R-loop formation, rather than its stability, is critical for pilin antigenic variation (13).

There are several limitations of this work, as all experiments were performed in one strain background, a highly laboratory-adapted strain engineered to facilitate the analysis of pilin Av. As such, this system may not fully reflect the range of genomic and regulatory variation present in contemporary Gc isolates. Differences in sequence context, replication dynamics, or DNA replication and repair activities are likely to influence how strand-specific obstacles, such as CRISPRi binding, affect genome stability and recombination. Thus, while our data support a model in which a localized, strand-specific physical obstacle is sufficient to trigger pilin Av, the extent to which this mechanism operates in physiological conditions across diverse gonococcal strains remains to be determined.

Pilin antigenic variation depends on the formation of transcription-associated R-loops and G4 structures at the *pilE* locus (14, 15). However, our results show that pilin Av still occurs when these native nucleic acid structures are not required. We therefore conclude that these structures are not essential *per se*; rather, a localized, strand-specific physical obstacle is the critical feature that enables gene conversion at *pilE*. Under physiological conditions, this obstacle is likely provided by the native R-loop/G4 structures (16, 32), whereas in our system, Cascade binding and the associated R-loop are sufficient to support pilin antigenic variation. Collectively, these results suggest that a localized, strand-specific physical obstacle, rather than defined nucleic acid structures, is a key determinant promoting pilin Av in Gc.

## MATERIALS AND METHODS

### Bacteria and strains

Strains and their details are listed in Table S1. All strains are FA1090 derivatives where most of the known variable genes with morphological phenotypes have been removed or mutated: the parental strain SM112 was an *opaA-K* deleted (Opaless) background (33), *pilC2* phase-locked “on” (34), and the 1-81-S2 pilin variant (35). The use of FA1090 pilin variant 1-81-S2 (25) ensured that the consistency of initial *pilE* sequences throughout the different experiments, which is important since different variants of the pilin can lead to different morphological aspects of the colony. The Type I-C CRISPRi machinery was introduced by the transformation of genomic DNA from non-targeting CRISPRi (23).

### Growth conditions

The strains were grown on Gonococcal (Gc) Medium Base (BD Difco) (36.25 g/L), agar (1.25 g/L), Kellogg Supplement I (22.2 mM glucose, 0.68 mM glutamine, and 0.45 mM cocarboxylase), and Kellogg Supplement II [1.23 µM Fe(NO_3_)_3_] (36). *Neisseria gonorrhoeae* was grown at 37°C at 5% CO_2_ for 30 h, with isopropyl β-D-1-thiogalactopyranoside (IPTG) added to the media to induce the Type I-C CRISPRi machinery.

### Spacer sequence design

CRISPR spacers that guide the Cascade complex to the target site were designed as previously documented (37). The consensus *Neisseria lactamica* PAM sequence (5′TTC′3 and 5′TTT′3) was found within the 100 bp upstream of the garP promoter. The PAM is 5′ to the spacer sequence, and the spacer sequence consists of the 35 bp immediately downstream of the PAM. The spacer guide RNA was introduced in the CRISPRi machinery as described by Geslewitz et al. (23). The primers used were, for the strain SM172, For (gtgtgttgattgggtcgGTTTCAACACACAGCCGC), and Rev (ccgataccccatgccaatTTTTTTTTCTAGATATCGCTCAATACTG), and for the strain SM174, For (cgataccccatgccaatGTTTCAACACACAGCCGC) and Rev (ggtgtgttgattgggtcgTTTTTTTTCTAGATATCGCTCAATACTG).

### *pilE* variant sequencing

To sequence *pilE* variants, blebs were sampled after 30 h of growth and re-isolated on plates without IPTG to avoid further *pilE* variation. A single colony was used for a PCR using the primers PilRBS (GGCTTTCCCCTTTCAATTAGGAG) and S3PA (CCGGAACGGACGACCCCG) (25). The PCR products were purified enzymatically using ExoSAP, and sent for Sanger sequencing at Azenta Life Science (Genewiz).

### Gonococcal transformation

Genetic transformation was performed by spotting the mix of 10 µL of plasmid DNA (~50 ng) and 10 µL of MgSO_4_ on a lawn of the recipient bacterial strain, on gonococcal base (GCB) plates containing IPTG. The plates were incubated for 18–20 h, and then the colonies that grew on the plasmid mix spot were isolated on GCB plates containing the antibiotic of interest. The colonies were selected on antibiotics twice before being checked.

### Digital qRT-PCR

Cells were treated with RNAprotect (Qiagen) prior to RNA extraction. Total RNA was isolated using an RNA extraction kit (Qiagen) and treated with DNase I for 2 h at 37°C to remove residual genomic DNA. RNA concentration was determined using the Quant-iT assay. Reverse transcription was performed using the QuantiTect Reverse Transcription Kit (Qiagen). Digital PCR was carried out using the QIAcuity EG system (Qiagen) according to the manufacturer’s instructions. Primers were designed using Primer3Plus. The primer sequences used were ACGCGTTAGAATAGGGTAGGT (forward) and GCCGGTTGCATAGAAACACC (reverse). Primer efficiency was validated by conventional PCR prior to digital PCR analysis. Housekeeping gene *rmp* was used as an internal control (Fig. S1) (38).

## Data Availability

The authors confirm that all supporting data, code, and protocols have been provided within the article and its supplemental material. The raw data generated and analyzed during this study are openly available in the PRISM repository at https://doi.org/10.18131/jzwk7-52026.

## SUPPLEMENTAL MATERIAL

Supplemental material mbio.01347-26-s0001.docx Supplemental figures and table.

ASM does not own the copyrights to Supplemental Material that may be linked to, or accessed through, an article. The authors have granted ASM a non-exclusive, world-wide license to publish the Supplemental Material files. Please contact the corresponding author directly for reuse.
