# Supplementary material for: CRISPRi targeting to the Neisseria gonorrhoeae R-loop or G4 mutant region restores pilin antigenic variation
Source: mBio. 2026 Jul 6;17(8):e01347-26. doi: 10.1128/mbio.01347-26 (PMC13463697; doi:10.1128/mbio.01347-26)
Supplement: Supplementary file 1 [file mbio.01347-26-s0001.docx]

# **Supplementary Data**

**Figure S1.** **CRISPRi inhibits the *gar* sRNA transcript.** The *gar* sRNA was quantified by digital RT‑PCR in the absence (–IPTG) or presence (+IPTG). Expression levels are reported as the *gar* sRNA /*rmp* ratio, with *rmp* used as the internal reference gene. **A.** Strain 172, G4-locked, CRISPRi targeting the leader strand. **B.** Strain 174, G4 locked, CRISPRi targeting the lagging strand. **C.** Strain 611, *gar* sRNA -10 promoter mutant, CRISPRi targeting the leader strand. **D.** Strain 614, *gar* sRNA -10 promoter mutant, CRISPRi targeting the lagging strand. Statistical significance was assessed using a Student’s t‑test.

Figure S2. **Alignment of *pilE* variants recovered from blebs of SM172 grown in the presence of IPTG.**
The first sequence corresponds to the reference *pilE* 1‑81‑S2 (GenBank: WBS96575.1). The first eight *pilE* sequences obtained from unpiliated colonies were intact, whereas the remaining thirty‑six sequences correspond to pilin antigenic variants for which the *pilS* donor sequences could be identified. The L‑pilin sequence was excluded for improved readability. Sequence alignment was performed using fast progressive FFT-NS-2 2 method with default parameters and visualized with Jalview (Waterhouse et al. 2009).

**Figure S3.** Growth of SM174 constructs on solid media, CFU/colony were measured in the presence or absence of 1mM IPTG after 30h of growth. Data points are presented as mean ± SD of three independent biological replicates; statistical significance was assessed using a Student’s t-test.

**Figure S4.**  Growth curves of the strain SM515 in GCBL medium with or without IPTG induction (black arrow), which activates the Type I‑C CRISPRi machinery. The strain SM515 is a previously published strain (Geslewiz et al. 2023), which CRISPRi machinery targets seven sites across the genome, three on the lagging strand and four on the leading strand. Data points are presented as mean ± SD of three independent biological replicates.

Table S1. Strains used in this study

| Strain name | Description | Reference/Source |
| --- | --- | --- |
| N-1-60 | *Neisseria gonorrhoea* FA1090 G4-locked parental strain | Hu et al. 2022 |
| SM111 | Opa deficient, G4-locked strain | Criss et al. 2013 |
| CRISPRi- nontargeting | Opa deficient background with Type I-C CRISPRi gene loci, non-targeting spacer,  1-81-S2 *pilE* sequence | Geslewiz et al. 2023 |
| SM112 | SM111, *pilC* locked, G4-locked, 1-81-S2 *pilE* sequence | This study |
| SM175 | SM111, *pilC* locked, G4-locked with Type I-C CRISPRi gene loci, non-targeting spacer, 1-81-S2 *pilE* sequence | This study |
| SM172 | SM112, G4-locked with CRISPRi targeting leader strand | This study |
| SM174 | SM112, G4-locked with CRISPRi targeting lagging strand | This study |
| SM197 | SM172, Native G4/*gar* sRNA with CRISPRi targeting leader strand | This study |
| SM199 | SM174, Native G4/*gar* sRNA with CRISPRi targeting lagging strand | This study |
| SM84 | recA4 (*recA*::tetR) | Seifert et al. 1994 |
| SM215 | SM172 *recO*::Km | This study |
| SM219 | SM174 recO::Km | This study |
| SM201 | SM172 *recA4*::tet | This study |
| SM203 | SM174 *recA4*::tet | This study |
| SM384 | SM172 + complementary *garP*-G4 sequence KmR | This study |
| SM388 | SM172 + reversed complement *garP* -G4 sequence KmR | This study |
| SM393 | SM172 + native *garP*-G4 KmR | This study |
| SM396 | SM172 + antisense *garP* -G4 KmR | This study |
| SM411 | SM174 + native *garP*-G4 KmR | This study |
| SM415 | SM174 + antisense *garP* -G4 KmR | This study |
| SM419 | SM174 + complementary *garP*-G4 sequence KmR | This study |
| SM423 | SM174 + reversed complement *garP* -G4 sequence KmR | This study |
| SM515 | N-1-60 genetic background with Type I-C CRISPRi gene loci, spacer 5 from *opa* multi-spacer array | Geslewiz et al. 2023 |
| SM610 | FA1090, 1-81-S2 *pilE* sequence, *garP-10* mutation unmarked | Prister et al. 2020 |
| SM611 | SM610 with CRISPRi targeting leader strand | This study |
| SM614 | SM610 with CRISPRi targeting lagging strand | This study |
